# Brain Metabolite Changes After Anodal Transcranial Direct Current Stimulation in Autism Spectrum Disorder

**DOI:** 10.3389/fnmol.2020.00070

**Published:** 2020-06-04

**Authors:** Narong Auvichayapat, Niramol Patjanasoontorn, Warinthorn Phuttharak, Chanyut Suphakunpinyo, Keattichai Keeratitanont, Orathai Tunkamnerdthai, Benchaporn Aneksan, Wanalee Klomjai, Wuttisak Boonphongsathian, Akkharawat Sinkueakunkit, Wiyada Punjaruk, Somsak Tiamkao, Paradee Auvichayapat

**Affiliations:** ^1^Department of Pediatrics, Faculty of Medicine, Khon Kaen University, Khon Kaen, Thailand; ^2^Department of Psychiatry, Faculty of Medicine, Khon Kaen University, Khon Kaen, Thailand; ^3^Department of Radiology, Faculty of Medicine, Khon Kaen University, Khon Kaen, Thailand; ^4^Department of Physiology, Faculty of Medicine, Khon Kaen University, Khon Kaen, Thailand; ^5^Department of Anesthesiology, Faculty of Medicine, Khon Kaen University, Khon Kaen, Thailand; ^6^Integrated Epilepsy Research Group, Faculty of Medicine, Khon Kaen University, Khon Kaen, Thailand; ^7^Faculty of Physical Therapy, Mahidol University, Salaya, Thailand

**Keywords:** autism spectrum disorder, transcranial direct current stimulation, brain metabolites, magnetic resonance spectroscopy, locus coeruleus

## Abstract

**Objectives:**

Previous research has provided evidence that transcranial direct current stimulation (tDCS) can reduce severity of autism spectrum disorder (ASD); however, the exact mechanism of this effect is still unknown. Magnetic resonance spectroscopy has demonstrated low levels of brain metabolites in the anterior cingulate cortex (ACC), amygdala, and left dorsolateral prefrontal cortex (DLPFC) in individuals with ASD. The aim of this study was to investigate the effects of anodal tDCS on social functioning of individuals with ASD, as measured by the social subscale of the Autism Treatment Evaluation Checklist (ATEC), through correlations between pretreatment and posttreatment concentrations of brain metabolites in the areas of interest (DLPFC, ACC, amygdala, and locus coeruleus) and scores on the ATEC social subscale.

**Methods:**

Ten participants with ASD were administered 1 mA anodal tDCS to the left DLPFC for 20 min over five consecutive days. Measures of the ATEC social subscale and the concentrations of brain metabolites were performed before and immediately after the treatment.

**Results:**

The results showed a significant decrease between pretreatment and immediately posttreatment in the ATEC social subscale scores, significant increases in N-acetylaspartate (NAA)/creatine (Cr) and myoinositol (mI)/Cr concentrations, and a decrease in choline (Cho)/Cr concentrations in the left DLPFC and locus coeruleus after tDCS treatment. Significant associations between decreased ATEC social subscale scores and changed concentrations in NAA/Cr, Cho/Cr, and mI/Cr in the locus coeruleus were positive.

**Conclusion:**

Findings suggest that beneficial effects of tDCS in ASD may be due to changes in neuronal and glia cell activity and synaptogenesis in the brain network of individuals with ASD. Further studies with larger sample sizes and control groups are warranted.

## Introduction

The abnormalities of brain functions in individuals with autism spectrum disorder (ASD) are not fully understood (Trottier et al., 1999). There is some evidence of a specific local increase in the thickness of the fusiform gyrus associated with face processing impairment in individuals with ASD (Schultz, 2005; Dziobek et al., 2010), as well as evidence of abnormal synaptic maturation, resulting in neurobiological connectivity defects (Levy et al., 2009). Several studies have proposed that decreased cortical plasticity may play an important role in ASD pathogenesis (Markram et al., 2007; Oberman et al., 2012, 2016). At present, there is no effective treatment for ASD, although behavioral therapy is a standard recommendation (Myers et al., 2007); however, the outcomes of this therapy are unsatisfactory. In severe cases with attention-deficit disorder, pharmacological therapies may cause adverse effects such as drowsiness, dry mouth, nausea, agitation, behavioral activation, and sleep problems (Oswald and Sonenklar, 2007).

Transcranial direct current stimulation (tDCS) is one of the non-invasive brain stimulation techniques that was shown to be effective in reducing severity of ASD (Schneider and Hopp, 2011; Amatachaya et al., 2014, Amatachaya et al., 2015; D’Urso et al., 2015; Gómez et al., 2017; English et al., 2018; Esse Wilson et al., 2018). In a previous tDCS study with individuals with ASD, low-voltage stimulation (1–2 mA) was applied via anodal electrode to the left dorsolateral prefrontal cortex (DLPFC) for 20–30 min, and a positive effect was found immediately after the treatment (Schneider and Hopp, 2011). Likewise, our previous study showed significant improvement on the Autism Treatment Evaluation Checklist (ATEC): total scores decreased from 67.25 pretreatment to 58 posttreatment (*p* < 0.001). We also found a significant decrease in the mean ATEC social subscale scores in the tDCS group (14.45) compared to the sham group (17.7) (*p* = 0.015) (Amatachaya et al., 2014, Amatachaya et al., 2015). A study in which cathodal 1-mA tDCS over the left DLPFC was applied, a significant decrease in symptoms of ASD lasting 1 month was found (Gómez et al., 2017). D’Urso et al. (2015) used 10 sessions of cathodal tDCS over the left DLPFC and found 26.7% reduction of the total Aberrant Behavior Checklist score in ASD. Taken together, this evidence suggests that tDCS could be effective for individuals with ASD. However, there were no clinical studies that investigate the mechanism of action of tDCS in reducing severity of ASD symptom.

Magnetic resonance spectroscopy (MRS) is a non-invasive technique for measuring biochemical changes in different organs including the brain. It is able to quantify steady-state metabolic levels of neurotransmitters such as N-acetylaspartate (NAA), which is a marker of axonal integrity; the small molecules such as choline (Cho) in the cell membrane composition, which is a marker for membrane turnover and reflects neuronal connection (Cudalbu et al., 2012); glutamine combined glutamate (Glx), an important excitatory neurotransmitter; myoinositol (mI), an osmolyte and astrocyte marker, which reflects synaptogenesis (Walecki et al., 2003; Kubas et al., 2012); and creatine (Cr), which is often used as an internal standard that normalizes other metabolites (Cudalbu et al., 2012). Previous studies that used MRS provided evidence of abnormal brain metabolites in individuals with ASD (Levitt et al., 2003; DeVito et al., 2007; Endo et al., 2007; Kleinhans et al., 2007; Bernardi et al., 2011; Bejjani et al., 2012; Mori et al., 2013).

The DLPFC is the brain area for which positive findings after anodal tDCS were reported. For example, Fujii et al. (2010) found that NAA/Cr was significantly decreased, whereas Cho/Cr was significantly increased in the left DLPFC in pediatric participants with ASD compared with control group participants (Fujii et al., 2010). Horder et al. (2014) reported lower concentrations of NAA and no significant differences in concentrations of Cho and Glx in the left DLPFC of adult participants with ASD compared with control group participants (Horder et al., 2014). They suggested that NAA/Cr deficits in the brain region responsible for executive functions may be associated with social and communication difficulties in ASD (Fujii et al., 2010; Shepherd and Freiwald, 2018). There were also reports showing low levels of Cho in the left inferior anterior cingulate cortex (ACC) (Levitt et al., 2003), low levels of Glx in the right ACC (Bernardi et al., 2011), and decreased NAA, Cho, and mI in the ACC(Goji et al., 2017). Other MRS studies of ASD reported low levels of NAA in the left amygdala (Mori et al., 2013) and low levels of NAA and Glx in the left frontal cortex (Kleinhans et al., 2007). Recently, there was a hypothesis that explored the possibility of explaining the symptoms found in ASD in terms of inefficient neuromodulation using the functioning of the locus coeruleus and norepinephrine as exemplars (London, 2018).

These evidences showed that there were many brain areas involved in determining the ASD core symptoms such as DLPFC, ACC, amygdala, and locus coeruleus. Magnetic resonance spectroscopy is a safe method that provides in-depth information about the abnormal brain metabolites in such brain areas in ASD. To the best of our knowledge, there is no study about the relationship between these metabolite changes after tDCS, so every type of brain metabolites might provide a preliminary investigation of biological changed following tDCS. We hypothesize that tDCS can modulate the brain function in ASD, and then the brain metabolite concentrations would change after tDCS treatment and the symptoms of ASD would decrease. Therefore, the primary objective of this study was to find out concentrations of brain metabolite changes in the areas of interest after anodal tDCS. The secondary objective was to study the associations between brain metabolite changes and the ATEC social subscale score changes.

## Materials and Methods

### Study Design

**FIGURE 1:**
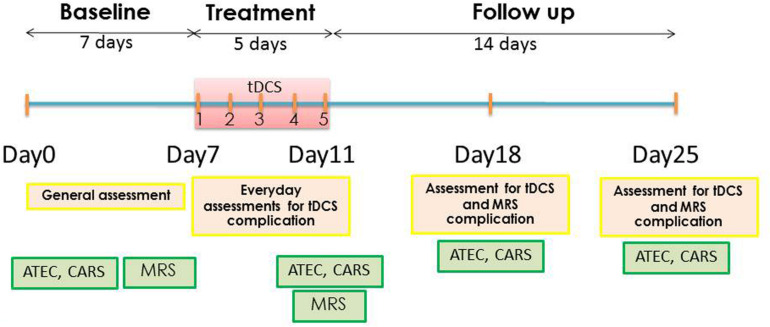
Schematic representation of experimental timeline.

This study was a pilot study composed of three phases: (1) baseline evaluation consisted of the baseline characteristic assessments, vital signs, ATEC, Childhood Autism Rating Scale (CARS), and baseline MRS; (2) treatment period consisted of 1 mA anodal tDCS for 20 min over five consecutive days and the post–tDCS treatment MRS, which was immediately performed after the fifth tDCS session; (3) the 2 weeks’ follow-up performed at days 7 and 14 after tDCS. The ATEC, CARS, adverse events of tDCS, and MRS were assessed at the first and second weeks after tDCS. Participants were asked to continue their routine medication regimen throughout the duration of the study. The schematic representation of timeline of the study is presented in Figure 1.

### Participant Recruitment and Informed Consent

Study participants were recruited by advertisement at the Pediatric Outpatient Children’s Psychiatric Clinic; Child Neurology Clinic; Child Development Clinic of Srinagarind Hospital, Faculty of Medicine, Khon Kaen University; and Khon Kaen Special Education Center Region 9, Thailand. The study procedures were described to caregivers who participating in the study by clinic physicians. Autism spectrum disorder diagnosis was confirmed by a child psychiatrist following a clinical review of the *Diagnostic and Statistical Manual of Mental Disorders, Fifth Edition* criteria (American Psychiatric Association, 2013).

The inclusion criteria were the same as in our previous study: (a) male participants with ASD; (b) age between 5 and 8 years; and (c) severe symptoms of ASD (CARS score ≥37). The exclusion criteria were the following: (a) having a pacemaker or metallic device; (b) severe neurological disorders such as brain tumor or intracranial infection; (c) drug abuse; (d) epilepsy; (e) skull defect; (f) use of herbal remedies or other alternative therapies; and (*g*) uncooperative parents and caregivers.

A routine brain magnetic resonance imaging (MRI) was performed on all participants to confirm the absence of organic disease, and MRS measurements were performed immediately afterward. Anesthesia was induced with 2 mg/kg intravenous propofol. After a laryngeal mask airway was inserted, participants received 2% sevoflurane and a 40:60 air/oxygen mixture for anesthesia maintenance at a total gas flow rate of 2 L/min. All participants were managed by the anesthetist team throughout the sedation period, and the procedures were performed following the guidelines for monitoring and management of pediatric patient sedation published by the American Academy of Pediatrics (Coté and Wilson, 2016).

The study was conducted in accordance with the Declaration of Helsinki and was approved by the Ethics Committee of Khon Kaen University (identifier no. HE 561188). The written informed consents were obtained from all participants and caregivers before participation.

### Measures

Two main outcomes were assessed in this study, the primary outcome was the ATEC social subscale, and the secondary outcome was the levels of brain metabolites in the brain areas of interest such as DLPFC, amygdala, ACC, and locus coeruleus. We also assessed vital signs, physical examination, and neurological examination to evaluate the possibility that tDCS might have adverse effects.

### Autism Treatment Evaluation Checklist

The ATEC is a questionnaire used to evaluate the effectiveness of treatments for individuals with ASD, which is completed by caregivers. It consists of four subscales: (1) speech/language/communication subscale (14 items; ceiling score 28); (2) social subscale (20 items; ceiling score 40); (3) sensory and cognitive awareness subscale (18 items; ceiling score 36); and (4) health/physical/behavior subscale (25 items; ceiling score 75). The total score ranges from 0 to 179; a higher score indicates worsening, whereas a lower score indicates improvement (Geier et al., 2012). The participants were assessed by the ATEC at baseline, immediately postsession on day 5, week 1, and week 2 after tDCS treatment.

Since there was research showing that NAA/Cr levels in the left DLPFC were related to social function (Fujii et al., 2010; Shepherd and Freiwald, 2018), we aimed to study associations between the change in the ATEC social subscale and brain metabolite posttreatment.

### Childhood Autism Rating Scale

The CARS is a well-established measure of ASD severity (Schopler et al., 1980; Chlebowski et al., 2010). The participants were evaluated using the CARS conducted by three investigators (N.P., C.S., and P.A.) who observed the participants and interviewed the parents and were unaware as to the treatment status of the participants. The details of the CARS evaluation were provided in as our previous study (Amatachaya et al., 2014, Amatachaya et al., 2015). The CARS is a 15-item behavioral rating scale developed to identify ASD and quantitatively describe its severity. The 15 items are as follows: relating to people, imitative behavior, emotional response, body use, object use, adaptation to change, visual response, listening response, other sensory responses, fear or anxiety, verbal communication, non-verbal communication, activity level, level and consistency of intellectual response, and general impressions (Rellini et al., 2004). Assessment was performed at baseline and immediately postsession on day 5, week 1, and week 2 after tDCS treatment.

### Brain Metabolite Levels

Our areas of interest were both DLPFC, both amygdalae, both ACC, and locus coeruleus. We used MRS to assess baseline and posttreatment levels of NAA, Glx, Cho, mI, and Cr in the regions of interest. Single-voxel proton magnetic resonance spectra were obtained from the areas of interest using a Philips Achieva 3.0 T (Philips Healthcare, Best, Netherlands) running Release 2.6.3.3 MR Workspace software. The procedures were performed under propofol-induced sedation to ensure that participants would lie still for approximately 60 min while the data were collected. Single-voxel proton magnetic resonance spectra were acquired and quantified with LC Model to determine metabolite concentration ratios. Magnetic resonance spectroscopy voxels (2 cm × 2 cm × 2 cm) were positioned on the coronal, sagittal, and axial images of the DLPFC, amygdala, ACC, and locus coeruleus. The spectra used a point resolved spectroscopy (PRESS) sequentially with a repetition time of 2 s, short echo time of 35 ms, spectral width of 2,000 Hz, 1,024 time points, and partial water suppression. Shimming was performed using manufacturer-supplied shimming procedures. The analysis of the metabolite concentrations was performed using LC-Model (Stephen Provencher Inc., Oakville, ON, Canada). Levels of NAA, Glx, mI, Cr, and Cho were analyzed by fitting a linear combination of a basis set of metabolite model spectra to the data. The analyzing spectrum was set between 3.8 and 0.2 ppm with no eddy-current correction and water scaling. The metabolite concentrations were expressed as nm and ratios relative to Cr peak. The metabolite concentrations and metabolite-to-Cr ratios were determined in NAA, Cho, mI, and Glx spectra for each subject.

### Adverse Events and Safety

Although the electric current induced by tDCS is weak (1 mA well below the pain threshold), it was applied continuously for five sessions of 20 min each (over the course of 5 days). The participants were asked to report any adverse events as well as other signs and symptoms immediately after each stimulation session. The participants were also closely observed by physicians during the study sessions. They were also followed up for adverse effects 7 and 14 days after the treatment.

### Transcranial Direct Current Stimulation

Transcranial direct current stimulation was applied via 0.9% NaCl-soaked pair of surface sponge electrodes (35 cm^2^) and delivered through battery-driven power supply. The constant direct current stimulator had a maximum output of 10 mA (Model 1224-B; Soterix Medical, New York, NY, United States). The anode electrode was placed over the left DLPFC, which was located using the 10–20 international system of electrode placement. A 1-mA current was applied for 20 min once a day for five consecutive days during the treatment period. The cathode, or the reference electrode, was placed on the right shoulder contralateral to the anode. The current was applied gradually by increasing it until the necessary current level was reached and then decreasing it after the stimulation.

### Data Analysis

Analyses were performed using the Stata software, version 10.0 (StataCorp, College Station, TX, United States). Data are presented as mean and standard deviation. Because of small sample size, the normality test was considered, and we found that most data were non-normal distribution. Therefore, we used the non-parametric Wilcoxon signed-ranks test to evaluate differences between pretreatment and posttreatment. Pre and post social subscale scores of ATEC and CARS were calculated in percent change. We examined the associations between the differences in brain metabolite levels and the social subscale of the ATEC by computing Pearson correlation coefficients between pretreatment to posttreatment changes in the measures of these domains. The effect sizes were determined by the mean difference between pretreatment and posttreatment and then dividing the result by the pooled standard deviation (Cohen *d*). Data are presented as means and SD. *p* < 0.05 was considered significant.

## Results

Ten children with ASD were screened for possible participation between November 2014 and February 2016, and all met the study inclusion criteria. Six right-handed and four left-handed participants completed the entire protocol without any adverse events. The mean age of the participants was 6.60 years (SD = 0.84 years); the mean age at diagnosis was 29.1 months (SD = 6.03 months). The demographic data are presented in Table 1.

**TABLE 1:** Demographic data of participants (*n* = 10).

Age (years) (mean ± SD)	6.60 ± 0.84
Sex (male/female)	10/0
Age of diagnosis (months) (mean ± SD)	29.10 ± 6.03
Delivery	
Normal labor	5
Cesarean section	5
Brain structure	
Normal	8
Increased in cerebellar volume	1
Callosal hypogenesis, decreased in gray matter volumes in the left frnto-inferior parietal cortex, and increased gray matter volume in the right supramarginal gyrus	1
Handedness (right/left)	6/4
Medication	
None	7
Risperidone	2
Pyritinol, methylphennidate, and risperidone	1
Non-medical treatment	
Occupational therapy	1
Developmental stimulation, speech therapy	4
Developmental stimulation, occupational therapy	1
Developmental stimulation, speech therapy, and occupational therapy	4
Risk factor	
Idiopathic	8
Family history	2
Autism Treatment Evaluation Checklist (ATEC)	
ATEC language	8.1 ± 3.20
ATEC social	12.5 ± 7.85
ATEC health	12.0 ± 7.20
ATEC sensory and cognitive function	23.3 ± 6.09
ATEC total score	55.9 ± 5.89
Childhood Autism Rating Scale Score (CARS)	44.0 ± 3.42

### Autism Treatment Evaluation Checklist Scores

The changes on the ATEC subscale scores from baseline to immediately posttreatment revealed a decrease of social subscale scores from 12.5 to 10.7 (14.4% change, *p* = 0.023); health subscale scores from 12.0 to 10.5 (12.5% change, *p* = 0.010), language subscale scores from 8.1 to 7.4 (8.6% change, *p* = 0.102), and sensory and cognitive function subscales from 23.3 to 21.3 (8.6% change, *p* = 0.016), as well as in the total ATEC score from 55.9 to 45.9 (17.9% change, *p* = 0.011). Data of ATEC social subscale scores at immediately posttreatment, week 1, and week 2 after treatments are presented in Table 2.

**TABLE 2:** Change of ASD symptoms at immediately posttreatment, week 1, and week 2 after treatment (*n* = 10).

	Baseline	Immediately posttreatment			Week 1			Week 2		
				
	Mean ± SD	Mean ± SD	*P*-value	Percent changes	Mean ± SD	*P*-value	Percent changes	Mean ± SD	*P*-value	Percent changes
Autism Treatment Evaluation Checklist (ATEC) Social Subscale Scores	12.5 ± 7.85	10.7 ± 9.35	0.023*	14.4%	10.2 ± 8.94	0.010*	18.4%	10.2 ± 8.62	0.011*	18.4%
Childhood Autism Rating Scale Scores (CARS)	44 ± 3.42	41.3 ± 3.20	0.005*	6.14%	41.3 ± 3.20	0.005*	6.1%	41.45 ± 2.98	0.005*	5.7%

*Statistical significance by Wilcoxon Signed Ranks Test, *p* < 0.05

### Childhood Autism Rating Scale Scores

At day 5 of anodal tDCS, the average absolute difference of CARS score was 2.7 (SD = 0.75, SEM = 0.24). The average percent change of CARS score was 6.14% (3.5–8.75%) A significant decrease in symptoms of ASD from baseline to immediately posttreatment was found (*p* = 0.005). With respect to the effect size (Cohen *d*), the pretreatment to immediately posttreatment change in CARS score was 0.84 (clinical significance). Data of CARS scores at immediately posttreatment, week 1, and week 2 after treatment are presented in Table 2.

### Changes in Brain Metabolites

Data of metabolites concentration at the baseline, immediately posttreatment in the left DLPFC, right DLPFC, left ACC, right ACC, left amygdala, right amygdala, and locus coeruleus are shown in Table 3. To study the changes in brain metabolites in the seven areas of interest, in the left DLPFC, Wilcoxon signed-ranks tests revealed a significant increase in NAA/Cr (*p* = 0.016) and mI/Cr (*p* = 0.012) and significant decrease in Cho/Cr (*p* = 0.037) (Figure 3). The effect sizes associated with the changes in NAA/Cr, Cho/Cr, and mI/Cr were 0.86, 0.82, and 0.82, respectively (clinically significant). The effect size associated with the change in Glx/Cr was 0.10 (negative). In the locus coeruleus, Wilcoxon signed-ranks tests revealed significant increase in NAA/Cr (*p* = 0.005) and mI/Cr (*p* = 0.025), decrease in Cho/Cr (*p* = 0.003), but no significant change in Glx/Cr (*p* = 0.120) at the locus coeruleus, from baseline to posttreatment (Figure 4). The effect sizes associated with the changes in NAA/Cr, Cho/Cr, mI/Cr, and Glx/Cr were 1.6, 0.69, 0.81, and 0.59, respectively.

**TABLE 3:** Metabolites concentration in the areas of interest at baseline and immediately posttreatment (*n* = 10).

	Baseline brain metabolites (Mean ± SD)	Immediately posttreatment (Mean ± SD)	Absolute mean difference	*P*-value
**Left dorsolateral prefrontal cortex**				
NAA/Cr	1.6517 ± 0.09	1.7207 ± 0.07	0.069	0.016*
Cho/Cr	0.699 ± 0.11	0.6237 ± 0.07	–0.076	0.037*
mI/Cr	0.208 ± 0.05	0.3007 ± 0.05	0.092	0.012*
Glx/Cr	0.321 ± 0.04	0.3267 ± 0.06	0.005	0.575
**Right dorsolateral prefrontal cortex**				
NAA/Cr	1.6407 ± 0.08	1.6967 ± 0.09	0.056	0.074
Cho/Cr	0.6377 ± 0.12	0.6167 ± 0.08	–0.021	0.171
mI/Cr	0.245 ± 0.03	0.2467 ± 0.08	0.001	0.646
Glx/Cr	0.3289 ± 0.02	0.3337 ± 0.02	0.004	0.734
**Left anterior cingulate cortex**				
NAA/Cr	1.318 ± 0.07	1.3687 ± 0.18	0.050	0.376
Cho/Cr	0.8907 ± 0.12	0.8407 ± 0.06	–0.050	0.095
mI/Cr	0.5445 ± 0.06	0.5607 ± 0.10	0.016	0.724
Glx/Cr	0.2104 ± 0.05	0.257 ± 0.07	0.047	0.114
**Right anterior cingulate cortex**				
NAA/Cr	1.3397 ± 0.24	1.3007 ± 0.31	–0.039	0.604
Cho/Cr	0.7997 ± 0.18	0.7597 ± 0.17	–0.040	0.114
mI/Cr	0.4897 ± 0.10	0.3947 ± 0.13	–0.095	0.097
Glx/Cr	0.2387 ± 0.05	0.2347 ± 0.08	–0.004	0.882
**Left amygdala**				
NAA/Cr	1.2997 ± 0.09	1.3327 ± 0.13	0.033	0.430
Cho/Cr	1.3007 ± 0.11	1.3597 ± 0.12	0.059	0.189
mI/Cr	0.4227 ± 0.15	0.4707 ± 0.09	0.048	0.192
Glx/Cr	0.2587 ± 0.08	0.2517 ± 0.09	–0.007	0.641
**Right amygdala**				
NAA/Cr	1.3117 ± 0.19	1.3067 ± 0.12	–0.005	0.948
Cho/Cr	0.8907 ± 0.19	0.9107 ± 0.09	0.020	0.703
mI/Cr	0.4307 ± 0.14	0.4567 ± 0.09	0.026	0.466
Glx/Cr	0.2487 ± 0.02	0.2547 ± 0.03	0.006	0.199
**Locus coeruleus**				
NAA/Cr	1.8497 ± 0.08	1.9917 ± 0.11	0.142	0.005*
Cho/Cr	1.2747 ± 0.09	1.2097 ± 0.09	0.065	0.003*
mI/Cr	0.5357 ± 0.08	0.6027 ± 0.08	0.067	0.025*
Glx/Cr	0.2257 ± 0.14	0.3557 ± 0.28	0.130	0.120

Absolute mean difference = mean of metabolite concentrations at posttreatment minus pretreatment. *Statistical significance by Wilcoxon signed-ranks test, *p* < 0.05.

**FIGURE 2:**
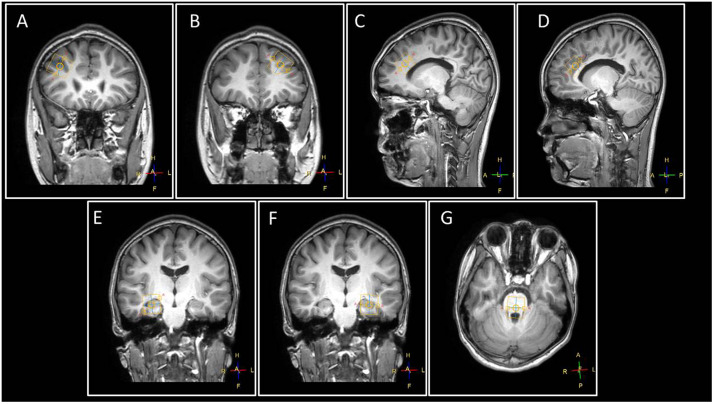
The voxels were placed at **(A)** left DLPFC, **(B)** right DLPFC, **(C)** right ACC, **(D)** left ACC, **(E)** right amygdala, **(F)** left amygdala, and **(G)** locus coeruleus. The yellow box indicates the location of a single voxel in the areas of interest. Abbreviations: A, anterior; F, feet; H, head; L, left; P, posterior; R, right.

**FIGURE 3:**
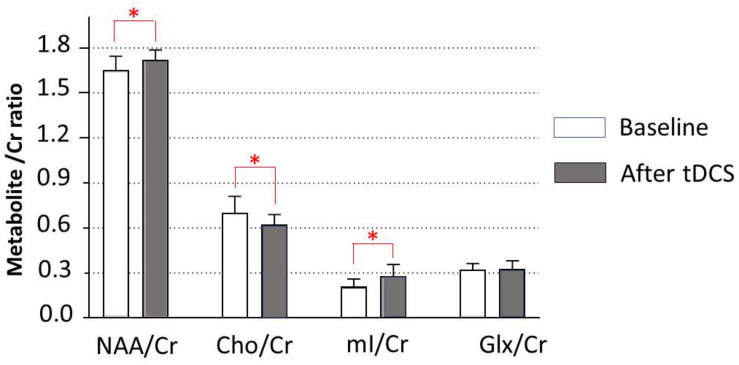
Metabolite changes in the left dorsolateral prefrontal cortex. Data are presented as means of NAA/Cr, Cho/Cr, mI/Cr, and Glx/Cr concentrations, compared between baseline (before tDCS) and after a 5-day tDCS treatment (day 5). Vertical lines represent SD. ^∗^Represents a significant difference; ^∗^*p* < 0.05.

**FIGURE 4:**
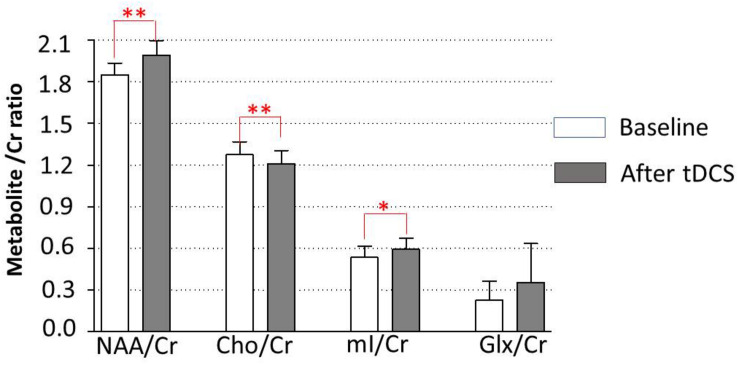
Metabolite changes in the locus coeruleus. Data are presented as means of NAA/Cr, Cho/Cr, mI/Cr, and Glx/Cr concentrations, compared between baseline (before tDCS) and after a 5-day tDCS treatment (day 5). Vertical lines represent SD. ^∗^Represents significant difference; ^∗∗^*p* < 0.01, ^∗^*p* < 0.05.

### The Associations Between the ATEC Social Subscale and Metabolite Changes

To study the associations between the social subscale of the ATEC and metabolite changes in the seven areas of interest, we computed a series of Pearson correlation coefficients. Our data showed significant associations between increased NAA/Cr and decreased ATEC social subscale (*r* = −0.684; *p* = 0.015), decreased Cho/Cr and decreased ATEC social subscale (*r* = 0.780; *p* = 0.004), and increased mI/Cr and decreased ATEC social subscale (*r* = −0.872; *p* = 0.001) in the locus coeruleus. The associations between the ATEC social subscale and metabolite changes in other areas of interest are shown in Table 4.

**TABLE 4:** Association between metabolite changes in the areas of interest and decreased ATEC social subscale scores (*n* = 10).

Brain metabolites	Left dorsolateral prefrontal cortex		Right dorsolateral prefrontal cortex		Left anterior cingulate cortex		Right anterior cingulate cortex		Left amygdala		Right amygdala		Locus coeruleus	
							
	Correlation coefficient (r)	*p*	Correlation coefficient (r)	*p*	Correlation coefficient (r)	*p*	Correlation coefficient (r)	*p*	Correlation coefficient (r)	*p*	Correlation coefficient (r)	*p*	Correlation coefficient (r)	*p*
NAA/Cr	−0.373	0.144	−0.273	0.223	−0.167	0.322	−0.212	0.278	0.251	0.242	−0.237	0.255	−0.684	0.015*
Cho/Cr	−0.375	0.143	−0.370	0.146	0.405	0.123	0.292	0.206	−0.540	0.053	0.376	0.142	0.780	0.004*
mI/Cr	−0.330	0.176	0.355	0.157	−0.110	0.382	−0.396	0.129	−0.222	0.269	−0.098	0.393	−0.872	0.001*
Glx/Cr	−0.327	0.178	−0.002	0.498	0.140	0.350	0.287	0.211	−0.214	0.276	0.146	0.344	0.089	0.403

Mean difference = Mean of metabolite concentrations at posttreatment minus pretreatment; *Statistical significance by Wilcoxon signed ranks test, p < 0.05.

## Discussion

Our study confirmed that tDCS had 17.9% reduction of ASD core symptoms assessed by ATEC total scores. The preliminary investigation of biological change following tDCS showed positive effect in the left DLPFC and the locus coeruleus. The significant associations between decreased ATEC social subscale and NAA/Cr, Cho/Cr, and mI/Cr concentration changes in the locus coeruleus were positive. N-acetylaspartate/Cr was tentatively increased in the right DLPFC. However, there were no significant changes in other brain metabolites either in the right DLPFC or in the left and right ACC and amygdala after the tDCS treatment.

Our study also showed clinical significant decrease in CARS scores from 44.0 to 41.30 (6.14% change), corresponding to the improvement of social–emotional understanding, emotional response, visual response, and verbal communication. The outcomes were similar to our previous study, which also revealed a significant decrease in CARS score from pretreatment to posttreatment. The effect of tDCS was maintained for 7 days in participants with the active tDCS condition relative to those in the sham tDCS condition (Amatachaya et al., 2014). Moreover, there was a report that showed a higher rate of chance corrected agreement (sensitivity of 0.89) between the CARS and clinical judgment for ASD (Chlebowski et al., 2010). The mechanisms of improved clinical outcomes of ASD after tDCS are not fully understood (Schneider and Hopp, 2011; Amatachaya et al., 2014, Amatachaya et al., 2015; D’Urso et al., 2015; Gómez et al., 2017). In this study, we surveyed the seven brain areas that have evidence related to ASD. We found changes in brain function of two parts, for example, the left DLPFC and the locus coeruleus detected by MRS, in which the brain metabolite changes were in the same pattern.

N-acetylaspartate is an amino acid derivative found exclusively in the central nervous system (CNS) neurons. It is considered as a marker of global neuronal health and attenuation (Verma et al., 2016). Lower levels of NAA are found in a variety of CNS disorders that cause neuronal cell destruction (Gonen et al., 2000; Rigotti et al., 2007) including ASD (Fujii et al., 2010). In general, NAA level quantified with ^1^H-MRS is thought to reflect neural density and indicate neuronal integrity and metabolism (Ford and Crewther, 2016). The decrease in parietal axon density and marked decrease in white matter NAA in children with ASD are associated with deficits in social functioning and memory (Levitt et al., 2003). Similarly, reduced NAA/Cr in the ACC is associated with poor social functioning (Fujii et al., 2010).

The results of this study support those of the previous study that NAA/Cr was lower in children with ASD (Fujii et al., 2010). The exact mechanism of increase in NAA after tDCS is not yet known. However, there is a possibility that the anodal tDCS can generally augment the neuronal excitability, and the current induces a sustainable response in the form of a long-term potentiation such as plasticity (Auvichayapat and Auvichayapat, 2011; Pelletier and Cicchetti, 2015).

Measured cortical Cho levels by ^1^H-MRS indicate membrane phosphatidylcholine breakdown. Choline levels in children are generally reduced, particularly in cortical gray matter, temporal regions, and the left thalamus, suggesting a decrease in membrane phospholipid turnover. In children with ASD, Cho/Cr level is reported to be increased. It represents abnormal regional increased membrane phospholipid turnover (Vasconcelos et al., 2008). Our result revealed a decrease in Cho/Cr after anodal tDCS treatment. This finding may suggest that tDCS could decrease regional membrane phospholipid breakdown (Pelletier and Cicchetti, 2015).

Myoinositol is thought to be a marker of astrocytes and plays an important role in glial cell proliferation. It also maintains cell metabolism and signaling as an intracellular postreceptor second messenger system. This second messenger system is linked to several receptors including glutamate receptors in the CNS. At present, there is no report of mI/Cr level in children with ASD (Ford and Crewther, 2016). However, our study showed an increase in mI/Cr as well as a positive association between the increased mI/Cr and decreased ATEC social subscale score after tDCS over the left DLPFC. It may be postulated that tDCS could reduce severity of ASD by increased synaptogenesis at the stimulation site (presynaptic neuron) or increased neuronal signaling to other brain areas (postsynaptic neuron) (Pelletier and Cicchetti, 2015).

There is still a lack of evidence about Glx concentration in individuals with ASD. Glutamate is involved in neurotransmitter regulation and detoxification. Reduced Glx in the ACC may relate to executive function deficits such as decision-making, impulse control, empathy, and emotion. However, our study showed no significant change in Glx/Cr after tDCS. This is compatible with a study that showed no effect of anodal tDCS on Glx/Cr levels in the left posterior superior temporal gyrus (Dwyer et al., 2018). This negative finding could be postulated that *N*-methyl-D-aspartate receptor and enzyme synthesis, which modulate the availability of neuronal, glial, and synaptic glutamate and glutamine, go undetected in ^1^H-MRS, thus inherently affecting the level of detected Glx (Ford and Crewther, 2016), or the Glx concentration did not change after anodal stimulation (Stagg et al., 2009).

Changes in brain function in the area of interest that were far away from the stimulation site should be explained by two concepts. First is the putative molecular mechanism of tDCS. Direct current electrical field (DCEF) can guide neural cell migration, growth cone direction, differentiation, and metabolism as a phenomenon called electrotaxis (Pelletier and Cicchetti, 2015). It is suspicious why a change has been found at the locus coeruleus, the most far away from the stimulation site. This can be explained by the greater total number of neurite branches found at the area near the cathode (McCaig et al., 2005). In this study, our cathode electrode was placed at the right shoulder, in which the locus coeruleus was the most nearby. Other areas of interest were far away from the cathode than the locus coeruleus, so the putative molecular mechanism of tDCS might occur difficultly and found the negative result for the brain metabolite changes. Second, there was more connectivity of the left DLPFC to locus coeruleus than the left DLPFC to other areas of interest.

Much of the recent research on ASD has focused not only on the brain structures but also on neuronal connectivity, and both underconnectivity and overconnectivity were reported based on connection lengths (London, 2018). The apparent abnormal neuronal connectivity in individuals with autistic is not fully understood. In general, the core symptoms of ASD are impaired social communication and restricted and repetitive behaviors. Neuromodulatory changes in ASD are suspected of involving many brain regions such as the frontal, temporal, and temporoparietal lobe, insular cortex, ACC, locus coeruleus, and limbic system (London, 2018; Shepherd and Freiwald, 2018). Input from a small number of neuromodulatory cells can abruptly interrupt the activity of neural networks and reorganize the elements into new functional networks; a single neuron can participate in several networks, and a single anatomical network can mediate multiple functions (London, 2018).

After stimulation at the left DLPFC, the results of our experiment showed a significant change in brain metabolites in the locus coeruleus.

The locus coeruleus is a brain area that receives inputs from a number of other brain regions including the prefrontal cortex; this connection is constant and excitatory and increases in strength with raised activity level of the individual (Ford and Crewther, 2016). The change in brain metabolites in the locus coeruleus in our study could be explained by the mechanism found in the DLPFC. The changes in the locus coeruleus may be a result of the changes in presynaptic neurons in the DLPFC. However, the associations between decreased scores on the ATEC social subscale and metabolite changes in the locus coeruleus were more positive than those in the left DLPFC. However, at present, there is little evidence of the locus coeruleus’ role in ASD. Nevertheless, there is evidence that atypical noradrenergic activity in the locus coeruleus is the underlying mechanism of aberrant attentional function in children with ASD (Bast et al., 2018). Thus, it is possible that the function of the locus coeruleus may be important in determining severity of ASD.

Taken together, changes in brain metabolites in the locus coeruleus after anodal tDCS can imply that the stimulation over the left DLPFC not only causes neuromodulation in the stimulated area but also modulates the locus coeruleus, the long-distance connectivity, which is impaired in individuals with ASD. The increased NAA/Cr and mI/Cr and decreased Cho/Cr in the locus coeruleus can reflect the mechanism of neuromodulation by increased neuronal integration, connectivity (Cudalbu et al., 2012), and synaptogenesis (Walecki et al., 2003; Kubas et al., 2012).

## Limitations

The present study has some limitations that should be considered when interpreting the results. First, as a pilot study, it had a small sample size that has been unable to detect significant effects in all areas of interest. Therefore, the findings presented here should be viewed as tentative and in need for replication by studies with larger samples. Second, it lacks a control or sham group. Further study using a control group would also be useful.

## Summary and Conclusion

To the best of our knowledge, this is the first study that addresses the beneficial effects of anodal tDCS on amelioration of ASD core symptoms. The study provides a preliminary investigation of biological changes following tDCS and the statistical changes of brain metabolites found in the left DLPFC and locus coeruleus, while there were no brain metabolite changes for the right DLPFC, left amygdala, right amygdala, left ACC, and right ACC. Increased NAA/Cr represents increase in neuronal function, increased mI/Cr represents increased glial cell proliferation and synaptogenesis, and decreased Cho/Cr represents the decreased cell membrane breakdown. The mechanism by which anodal tDCS improves the symptoms of ASD may related to the changes of these brain metabolites. However, a study with a larger sample size and a control group is warranted.

## Data Availability Statement

The raw data supporting the conclusions of this article will be made available by the authors, without undue reservation.

## Ethics Statement

The studies involving human participants were reviewed and approved by the Ethics Committee of Khon Kaen University (Identifier number: HE 561188). Written informed consent to participate in this study was provided by the participants’ legal guardian/next of kin.

## Author Contributions

PA, NA, and ST contributed conception and design of the study. BA, WK, and WP organized the database. OT and PA performed the statistical analysis. PA and KT wrote the first draft of the manuscript. PA, NP, and CS assessed autistic severity and abnormal behaviors. WP assessed brain MRI and MRS. WB was an MRS technician. AS was an anesthesiologist. PA, KT, and NA wrote sections of the manuscript. All authors contributed to manuscript revision, read and approved the submitted version.

## Conflict of Interest

The authors declare that the research was conducted in the absence of any commercial or financial relationships that could be construed as a potential conflict of interest.
